# BacSp222 bacteriocin as a novel ligand for TLR2/TLR6 heterodimer

**DOI:** 10.1007/s00011-023-01721-3

**Published:** 2023-03-25

**Authors:** Justyna Śmiałek-Bartyzel, Monika Bzowska, Renata Mężyk-Kopeć, Marcin Kwissa, Paweł Mak

**Affiliations:** 1 Doctoral School of Exact and Natural Sciences, Jagiellonian University, Łojasiewicza 11 St., 30-348 Kraków, Poland; 2 Department of Analytical Biochemistry, Faculty of Biochemistry, Biophysics and Biotechnology, Jagiellonian University, Gronostajowa 7 St., 30-387 Kraków, Poland; 3 Department of Cell Biochemistry, Faculty of Biochemistry, Biophysics and Biotechnology, Jagiellonian University, Gronostajowa 7 St., 30-387 Kraków, Poland; 4 Pritzker School of Molecular Engineering, University of Chicago, 5640 South Ellis Ave., Chicago, IL 60637 USA

**Keywords:** BacSp222 bacteriocin, FPR, Inflammation, LTA, PAMP, TLR2/TLR6

## Abstract

**Objective and design:**

BacSp222 bacteriocin is a bactericidal and proinflammatory peptide stimulating immune cells to produce selected cytokines and NO in NF-ĸB dependent manner. This study aims to identify the receptor which mediates this activity.

**Methods:**

We applied fluorescently labeled BacSp222 and a confocal microscopy imaging to analyze the direct interaction of the bacteriocin with the cells. Reporter HEK-Blue cells overexpressing human toll-like receptors (TLR2, TLR4, TLR5 or TLR2/TLR1 and TLR2/TLR6 heterodimers) were stimulated with BacSp222, and then the activity of NF-ĸB-dependent secreted embryonic alkaline phosphatase (SEAP) was measured. In turn, formylated peptide receptor (FPR) or TLR2 antagonists were used to verify bacteriocin-stimulated TNF production by murine monocyte-macrophage cell lines.

**Results:**

BacSp222 undergoes internalization into cells without disturbing the cell membrane. FPR antagonists do not affect TNF produced by BacSp222-stimulated murine macrophage-like cells. In contrast, BacSp222 stimulates NF-ĸB activation in HEK-Blue overexpressing TLR2 or TLR2/TLR6 heterodimer, but not TLR2/TLR1, TLR4 or TLR5 receptors. Moreover, TLR2-specific antagonists inhibit NF-ĸB signaling in BacSp222-stimulated HEK-Blue TLR2/TLR6 cells and reduce TNF release by BacSp222-treated RAW 264.7 and P388.D1.

**Conclusions:**

BacSp222 is a novel ligand for TLR2/TLR6 heterodimer. By binding TLR complex the bacteriocin undergoes internalization, inducing proinflammatory signaling that employs MyD88 and NF-ĸB pathways.

**Supplementary Information:**

The online version contains supplementary material available at 10.1007/s00011-023-01721-3.

## Introduction

The human microbiota was once called a “forgotten organ” due to its underappreciated role in physiology and maintaining the health of our organism [1]. The human symbiotic and commensal bacteria function as a kind of physical barrier, protecting us against foreign pathogens through competitive exclusion and the production of antimicrobials [2]. Thanks to the gut microbiota, our organism benefits from the ability to metabolize certain carbohydrates and biosynthesize exogenous vitamins and short-chain fatty acids [3]. The bacterial microbiota is also essential for developing the mucosal immune system. Germ-free laboratory animals exhibit shorter lifespans, increased susceptibility to pathogens, are severely deficient in K and B12 vitamins, lack natural antibodies, have deficits in gut-associated lymphoid tissues, and aberrant natural killer T-cells, as well as show increased morbidity in models of inflammatory and allergic diseases [4, 5]. Of course, bacteria also have another face related to diseases. After the injury, microbes can colonize normally sterile tissues and cause acute infections and inflammation; bacterial pathogens and commensals are also responsible for many classical systemic infectious diseases. Moreover, alteration in the composition or development of the human microbiota (so-called dysbiosis of microbiota), as well as a disturbance in molecular communication between the host and commensal bacteria, is associated with many inflammatory and metabolic diseases, neurological disorders and even carcinogenesis [2, 5, 6].

All the above phenomena emphasize the critical role of mechanisms that control the number of cells in bacteria populations as well as the inflammatory reaction of the host. At the bacteria level, three large groups of mechanisms regulate the size and diversity of cell populations in particular habitats: the quorum sensing phenomenon, the production of antibiotics, and the excretion of bacteriocins. Quorum sensing is an intercellular communication system of bacteria, mediated by small diffusible signaling molecules (so-called autoinducers), and able to regulate different genes expression in response to cell population density [7]. In turn, antibiotics are low-molecular products of the secondary metabolism of bacteria, in low concentrations capable of killing or inhibiting the growth of other microorganisms [8]. The third large group of molecules able to control the bacterial populations’ density and diversity are bacteriocins. They are peptides and proteins that kill or inhibit the growth of similar or closely related bacterial strains [9]. All bacteriocins are synthesized on ribosomes, and this fact fundamentally distinguish them from peptide antibiotics-which are synthesized on specialized enzyme complexes, called non-ribosomal peptide synthetases (NRPS) [10]. It is estimated that almost every bacterial specie can produce at least one kind of bacteriocins, and, assuming such widespread prevalence, these molecules appear to be critical factors influencing the proper microbiome composition of the skin, mucous membranes, gastrointestinal and urogenital tracts [11]. In recent years our studies have focused on bacteriocins produced by staphylococci-widespread residents and opportunistic pathogens of the skin and mucous membranes of humans and many animals. One of such strains, zoonotic *Staphylococcus pseudintermedius* strain 222, produces BacSp222 bacteriocin—a linear 50-amino acids-long peptide belonging to leaderless subclass IId bacteriocins and to structural four-helix bundle group of staphylococcins [12–14]. As befits a typical bacteriocin, at micromolar and sub-micromolar concentrations, BacSp222 effectively kills a wide range of Gram-positive bacteria. But the BacSp222 molecule attracts our special attention because it breaks the convention related to the strictly bactericidal activities of bacteriocins and proves that they may also serve as factors able to modulate the host's inflammatory reaction. The studies performed on mouse monocyte/macrophage-like cell lines and on human neutrophils demonstrated the proinflammatory activity of BacSp222. At nanomolar concentrations, BacSp222 activated NF-κB-a major transcription factor controlling the inflammatory response. In murine macrophage-like cell line, BacSp222 exposure resulted in increased secretion of TNF, MCP-1, and IL-1α, as well as enhanced nitric oxide (NO) production by inducible NO synthase (iNOS). In human neutrophils, BacSp222 upregulated IL-8 production but, on the other hand, did not induce reactive oxygen species (ROS) production nor neutrophil extracellular traps (NETs) formation [15].

The present study concerns the identification of cellular receptors responsible for sensing BacSp222 by eukaryotic cells. We focused on two particular classes of receptors, formyl peptide receptors (FPRs) and Toll-like receptors (TLRs), present on various innate immune cells and able to detect bacterial-derived pathogen-associated molecular patterns (PAMPs), as well as to induce inflammatory response [15, 16]. Our experiments were conducted using murine monocyte/macrophage-like cell lines and engineered human embryonic kidney (HEK) reporter cells with stable overexpression of different human TLR genes. We verified the ability of murine macrophage-like cells to internalize BacSp222, studied the influence of specific antagonists of FPRs and TLRs on BacSp222 activity, and analyzed the interaction of BacSp222 with specific TLRs. Besides BacSp222 the experiments were performed also using its form chemically deprived of N-terminal formyl-Met residue (-fM-BacSp222), as well as the post-translationally modified form of bacteriocin, a suc-K20-BacSp222 peptide, produced by bacteria in response to particular environmental factors [14]. All peptides were highly purified and carefully verified for possible contamination of other bacterial-derived immunogens, such as lipopolysaccharides (LPS) or lipoteichoic acids (LTA). Such a fact distinguishes our work from other studies concerning the immunomodulatory potential of bacteriocins [17].

## Materials and methods

### Peptides and protein chemistry techniques

If not otherwise stated, all chemicals and materials used in this work were from Merck (Darmstadt, Germany) while all solutions were prepared using ultrapure, endotoxin-free water from Purelab Maxima system (ELGA LabWater, High Wycombe, United Kingdom).

BacSp222 bacteriocin, its succinylated form (suc-K20-BacSp222), as well as bacteriocin deprived of N-terminal formyl-methionine (-fM-BacSp222) were obtained and analyzed strictly as described in our previous work [15]. As is presented in the above-cited study, the purity (over 99%), homogeneity, and identity of the peptides were checked by an analytical reversed-phase high-pressure liquid chromatography (RP-HPLC) and mass spectrometry. At the same time, the possible contamination by a Gram-negative endotoxin, LPS, was excluded during an E-TOXATE assay.

Additionally and independently, the possible contamination of peptides by LPS and LTA (a Gram-positive endotoxin) was carefully verified by total phosphorus determination performed by a modified method originally developed by Huang and Zhang [18]. This method involves converting the total dissolved organic phosphorus (present in all forms of the mentioned bacterial endotoxins) into an inorganic phosphate, determined spectrophotometrically after subsequent reaction with a mixture of molybdate-antimony reagent. In the present study, this technique has been adapted to the microplate format as follows: 238 µL portions of appropriate peptide solutions or an LTA standard (LTA from *Staph. aureus*, cat. No L2515, Merck, Darmstadt, Germany) were pipetted into the wells of a 96-well flat-bottom polystyrene cell culture microplate (Nest, Wuxi, China) and mixed with 48 µL portions of K_2_S_2_O_8_ solution (2 g in 40 mL H_2_O). The wells were sealed tightly using a MicroAmp film (Applied Biosystems/Thermo, Waltham, MA, USA) and incubated overnight at 90 °C in a thermal cycler (model C1000 Touch, Bio-Rad, Hercules, CA, USA). After cooling down, the content of the wells was mixed with 71 µL of a 1:1 mixture of ascorbic acid solution (0.5 g in 50 mL H_2_O) and molybdate-antimony solution (0.12 g N_6_H_24_Mo_7_O_24_ · 4H_2_O, 46 mL H_2_O, 1.25 mL 98% w/v H_2_SO_4_, 2.5 mL 0.3% w/v K_2_C_8_H_4_O_12_Sb_2_ · 3H_2_O). After 8 min incubation at room temperature, the absorbance was read at 890 nm using a microplate reader (model Sunrise, Tecan, Männedorf, Switzerland).

The fluorescent labeling of BacSp222 on carboxyl groups has been carried out using a CF488A dye (cat. No SCJ4600015, Merck, Darmstadt, Germany). 1 mg of CF488A was dissolved in 1 mL of 0.2 M 2-(N-morpholino) ethanesulfonic acid (MES) pH 5.0, and to this solution successively was added 20 µL portion of BacSp222 solution (500 µg in water), and then 50 µL portion of 1-ethyl-3-(3-dimethylaminopropyl) carbodiimide hydrochloride solution (EDC-HCl, cat. No PG82079, Pierce/Thermo, Waltham, MA, USA, 5.14 mg in 0.2 M MES pH 5.0). The mixture was mixed at room temperature for 2 h. After this the labeled peptide has been purified by RP-HPLC chromatography using a Discovery Bio Wide Pore C8 4.6 × 250 mm column and two buffers: A, containing 0.1% (v/v) trifluoroacetic acid (TFA) as well as B, containing 0.07% TFA and 80% (both v/v) acetonitrile. The linear gradient 45% to 100% of buffer B was developed for 10 min, the flow rate was 1 mL/min, and the labeled peptide has been manually collected as a broad peak eluting at 9 to 13 min. The collected fraction has been evaporated using a vacuum centrifuge and dissolved in water. As verified by bactericidal radial diffusion assay as well as by NO release experiment on macrophage-like cells (data not shown) the bacteriocin labeled by CF488A at carboxyl groups (BacSp222-CF488) maintained its full biological activity.

The concentration of all peptides used in this study was determined by an amino acid analysis as described earlier [19].

### Eukaryotic cells used in experiments

Murine monocyte/macrophage RAW 264.7 cell line (ATCC TIB-71) and murine monocyte/macrophage P388.D1 cell line (ATCC CCL-46) were obtained from the American Type Culture Collection (Manassas, VA, USA). Human TLR2/NF-κB/SEAP (HEK-Blue hTLR2), TLR4/NF-κB/SEAP (HEK-Blue hTLR4), TLR5/NF-κB/SEAP (HEK-Blue hTLR5), TLR2+TLR1/NF-κB/SEAP (HEK-Blue hTLR2 and hTLR1), and TLR2+TLR1/NF-κB/SEAP (HEK-Blue hTLR2 and hTLR6) reporter HEK293 cells were purchased from InvivoGen (San Diego, CA, USA). The passages of all TLR overexpressing cells were from 3 to 8.

The cells were grown in an incubator, at 5% CO_2_, 37 °C, and > 95% humidity, in Dulbecco’s modified Eagle’s medium (DMEM) containing 4.5 g/L glucose (GIBCO, Paisley, UK) and 5% (v/v) fetal bovine serum (FBS, GIBCO, Paisley, UK), (for RAW 264.7 and P388.D1 cells) or in DMEM containing 4.5 g/L glucose, 10% (v/v) FBS, 50 units/mL penicillin, and 50 µg/mL streptomycin (GIBCO, Paisley, UK) (in case of TLR family overexpressing HEK293 cells).

### The interaction of BacSp222-CF488 with P388.D1 cells: confocal microscopy imaging of live cells

AxioObserver Z.1 inverted optical microscope with a laser scanning confocal module LSM 880 (Carl Zeiss, Germany) was used to analyze the interaction of BacSp222-CF488 with P388.D1 cells. First, the cells (2 × 10^6^ in the volume of 2 mL DMEM supplemented with 5% FBS) were seeded on a 12 mm Nunc glass base dish (Thermo Scientific, Rochester, NY, USA). After overnight culture and prior imaging, the medium was replaced with fresh DMEM. Then the cells were transferred to an incubation chamber of the microscope (under temperature and CO_2_ control) and stimulated with 1 µM BacSp222-CF488 or 1 µM BacSp222 (for 5 or 30 min). Next, the cells were gently rinsed with PBS, and 2 mL of PBS containing 1.73 µM sulforhodamine B was added. Imaging was performed using oil immersion and Plan-Neofular 40 × NA1.3 objective. The argon laser line of 488 was used for BacSp222-CF488 excitation, and emission in the range 493–556 nm was recorded as the green channel. For sulforhodamine, 561 nm was used for excitation, and 566–703 nm emission was recorded as the red channel. ImageJ 1.53c software (National Institutes of Health, Bethesda, MD, USA) was used for image processing.

### The interaction of BacSp222-CF488 with P388.D1 cells: confocal microscopy imaging of fixed cells

P388.D1 cells (2 × 10^6^ cells per well) were seeded in 1 mL of DMEM/5% (v/v) FBS on poly-l-lysine-coated glass coverslips placed in the wells of a 12-well plate and grown overnight. Next, the medium was replaced with a fresh DMEM. The cells were stimulated for 5 or 30 min with 1 µM BacSp222 or 1 µM BacSp222-CF488 and fixed in a 4% (v/v) solution of methanol-free formaldehyde (Thermo Fisher Scientific, Waltham, MA, USA) in PBS for 15 min at room temperature (RT). In the next step, the cells were washed 3 times with PBS, and the nuclei were stained with 4′,6-diamidyno-2-fenyloindol (DAPI, Thermo Fisher Scientific, Waltham, MA, USA) in the dark at RT for 15 min. Then the cells were washed with PBS, and the samples were mounted onto slides in ProLong Glass Antifade Mountant (Thermo Fisher Scientific, Waltham, MA, USA) and stored for 24 h in the darkness. The cells were observed using AxioObserver Z.1 inverted optical microscope with a laser scanning confocal module LSM 880 (Carl Zeiss, Germany). Imaging was performed using oil immersion and Plan-Neofular 40 × NA1.3 objective. The argon laser line of 488 was used for BacSp222-CF488 excitation, and emission in the range 495–630 nm was recorded as the green channel. For DAPI, 405 nm was used for excitation, and emission in the range 410–495 nm was recorded as the blue channel. ImageJ 1.53c software (National Institutes of Health, Bethesda, MD, USA) was used for image processing.

### Inhibition of FPR1 and FPR2 by selected antagonists

RAW 264.7 or P388.D1 cells were grown overnight on 96-well plates in 100 µL of DMEM enriched with 5% (v/v) FBS (the density of cells was 3 × 10^4^ cells/well). Next, the medium was replaced with fresh DMEM supplemented with 2% (v/v) FBS (control) or fresh DMEM supplemented with 2% (v/v) FBS containing: 50 µM WRW4 (Millipore Corporation, affiliated by MERCK, Germany) or 5 µM Boc-2 (MP-Bio, France), and the cells were incubated for 30 min. After incubation RAW 264.7 or P388.D1 cells were treated for 6 h with: (1) 50 µM WRW4, (2) 5 µM Boc-2, (3) 1 µM fMLP, (4) 1 µM WKYMVM, (5) 1 µM BacSp222, (6) 1 µM suc-K20-BacSp222, (7) 1 µM -fM-BacSp222, (8) 5 µM Boc-2 and 1 µM fMLP, (9) 50 µM WRW4 and 1 µM fMLP, (10) 5 µM Boc-2 and 1 µM WKYMVM, (11) 50 µM WRW4 and 1 µM WKYMVM, (12) 5 µM Boc-2 + 1 µM BacSp222, (13) 50 µM WRW4 + 1 µM BacSp222, (14) 5 µM Boc-2 + 1 µM suc-K20-BacSp222, (15) 50 µM WRW4 + 1 µM suc-K20-BacSp222, (16) 5 µM Boc-2 + 1 µM -fM-BacSp222, (17) 50 µM WRW4 + 1 µM -fM-BacSp222. Next, the media were collected for TNF concentration measurement using the ELISA test **(**ELISA MAX™ Standard Set Mouse TNF; Biolegend, San Diego, CA, USA) according to the protocol provided by the manufacturer. The viability of the cells was determined and analyzed using a MTT assay as described previously [15]. The absorbance was measured at 450 nm for ELISA and 545 nm for MTT assays using the Synergy H1 Hybrid plate reader controlled by Gene5 version 2.00.18 software (BIOTEK Instruments, Winooski, VT, USA).

### Stimulation of overexpressing TLR receptors cells

HEK-Blue hTLR2, HEK-Blue hTLR4, HEK-Blue hTLR5, HEK-Blue hTLR2 and hTLR1, as well as HEK-Blue hTLR2 and hTLR6, were seeded on a 96-well plate at density 2.5 × 10^4^ cells per well in 100 µL DMEM containing 10% (v/v) FBS, 50 units/mL penicillin, 50 µg/mL streptomycin (control) or in 100 µL DMEM containing 10% (v/v) FBS, 50 units/mL penicillin, 50 µg/mL streptomycin and (1) 5 × 10^7^ Heat Killed *Listeria monocytogenes* cells/mL (HKLM, InvivoGen, San Diego, CA, USA) or 10 ng/mL ultrapure LPS from *Escherichia coli* 0111:B4 (InvivoGen, San Diego, CA, USA), or 10 ng/mL ultrapure flagellin from *Bacillus subtilis* (InvivoGen, San Diego, CA, USA), or 10 ng/mL FSL-1 (InvivoGen, San Diego, CA, USA), or 20 µM CU-T12-9 (InvivoGen, San Diego, CA, USA) (the positive controls for HEK-Blue hTLR2, HEK-Blue hTLR4, HEK-Blue hTLR5, HEK-Blue hTLR2 and hTLR1, HEK-Blue hTLR2 and hTLR6, respectively), (2) 1 µM BacSp222, (3) 1 µM suc-K20-BacSp222, (4) 1 µM -fM-BacSp222. After 17 h, the media were collected for secreted embryonic alkaline phosphatase (SEAP) detection according to the protocol described in the section below. The viability of the cells was determined and analyzed using an MTT assay as described previously [15].

### Inhibition of human TLR2 and TLR6 heterodimer by the selected antagonists

HEK-Blue hTLR2 and HEK-Blue hTLR6 cells were seeded on a 96-well plate at density 2.5 × 10^4^ cells per well in 100 µL DMEM containing 10% (v/v) FBS, 50 units/mL penicillin, 50 µg/mL streptomycin (control) or in 100 µL DMEM containing 10% (v/v) FBS, 50 units/mL penicillin, 50 µg/mL streptomycin and (1) 20 µM sparstolonin B, (2) 200 µM TL2-C29 (InvivoGen, San Diego, CA, USA). After 1 h to each well was added 10 µL of (1) water (control), (2) FSL-1 (to a final concentration of 10 ng/mL), (3) BacSp222 (to a final concentration of 1 µM), (4) suc-K20-BacSp222 (to a final concentration 1 µM), or (5) -fM-BacSp222 (to a final concentration 1 µM). After 17 h, the media were collected for SEAP detection according to the protocol described below. The viability of the cells was determined using an MTT assay as described previously [15].

### Assay for detection of secreted embryonic alkaline phosphatase (SEAP)

Activation of NF-κB upon TLRs stimulation was analyzed by measurement of SEAP activity secreted by the cells to the media in response to bacteriocins. 10 µL of culture media were collected from the cells and transferred to a 96-well plate containing 90 µL Cell Culture Medium for SEAP Detection (InvivoGen, San Diego, CA, USA). The plate was incubated at 37 °C for 1 h, and the absorbance was measured at 620 nm using a microplate reader Synergy H1 Hybrid plate reader controlled by Gene5 version 2.00.18 software (BIOTEK Instruments, Winooski, VT, USA).

### Inhibition of murine TLR2 by the selected antagonists

RAW 264.7 cells were grown overnight on 96-well plates in 100 µL of DMEM enriched with 5% (v/v) FBS (the density of the cells was 3 × 10^4^ cells/well), while P388.D1 cells were grown overnight on 48-well plates in 200 µL of DMEM enriched with 5% FBS (v/v) (the density of the cells was 6 × 10^4^ cells/well). Next, the medium was replaced with 100 µL fresh DMEM with 2% (v/v) FBS or fresh DMEM with 2% (v/v) FBS containing: (1) 6 µM sparstolonin B or (2) 200 µM TL2-C29 (InvivoGen, San Diego, CA, USA). After 30 min, the medium was replaced again with fresh DMEM supplemented with 2% (v/v) FBS or DMEM supplemented with 2% (v/v) FBS and containing (1) 2 µg/mL LTA, (2) 1 µM BacSp222, (3) 1 µM suc-K20-BacSp222, (4) 1 µM -fM-BacSp222*,* (5) 6 µM sparstolonin B, (6) 6 µM sparstolonin B and 2 µg/mL LTA, (7) 6 µM sparstolonin B and 1 µM BacSp222, (8) 6 µM sparstolonin B and 1 µM suc-K20-BacSp222, (9) 6 µM sparstolonin B and 1 µM -fM-BacSp222, (10) 200 µM TL2-C29, (11) 200 µM TL2-C29 and 2 µg/mL LTA, (12) 200 µM TL2-C29 and 1 µM BacSp222, (13) 200 µM TL2-C29 and 1 µM suc-K20-BacSp222, (14) 200 µM TL2-C29 and 1 µM -fM-BacSp222. After 6 h, the media were collected for TNF concentration analysis using the ELISA test described above.

### Analysis of NO production by the cells

RAW 264.7 cells were seeded on 96-well plates in 100 μL DMEM supplemented with 5% (v/v) FBS at 3 × 10^4^ cells/well density. After 16 h, the medium was replaced with fresh DMEM with 2% (v/v) FBS or DMEM containing 2% (v/v) FBS and LTA in the concentration range between 0,005 µg/mL to 2 µg/mL. Each stimulation was in the presence or absence of 10 ng/mL mouse interferon γ (IFN-γ, Biolegend, San Diego, CA, USA). The nitrate levels were measured as described previously [15].

### Statistical analysis and data presentation

Experiments were performed with three independent replications. The data were presented as mean ± standard deviation (SD). The Statistica software (Tibco Software, version 13.3) was used to perform statistical analysis. The statistical significance of differences between the particular results was calculated by a one-way ANOVA with Tukey’s HSD (honestly significant difference) post hoc test and shown in figures as * for *p* < 0.05 and # for *p* < 0.001.

## Results

### BacSp222-CF488 undergoes internalization into P388.D1 cells

**Fig. 1:**
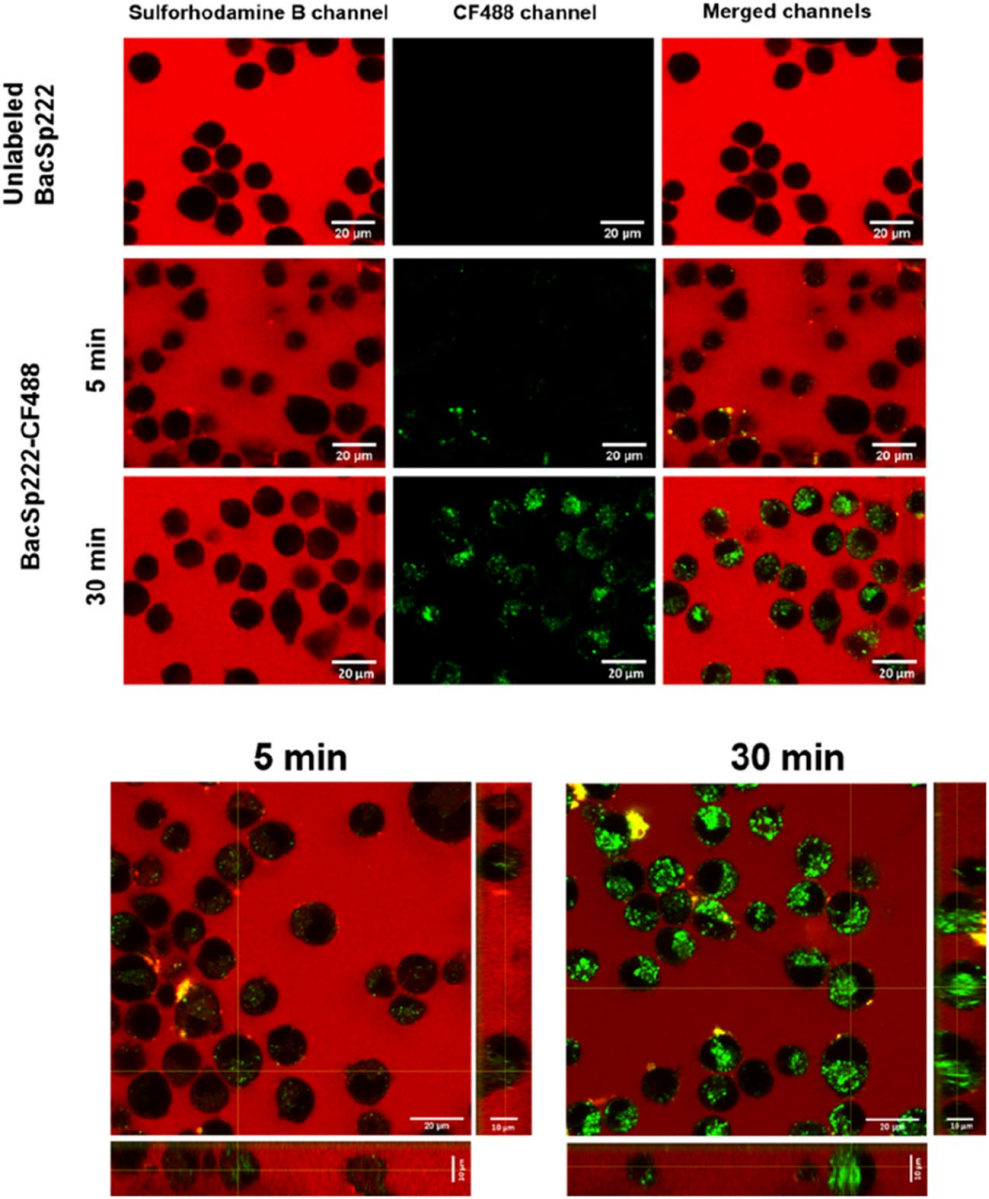
Interaction of fluorescently labeled BacSp222-CF488 with P388.D1 cells visualized using confocal microscopy. Unlabeled BacSp222 or BacSp222-CF488 was added to the cells for 5 or 30 min. After incubation, the cells were washed with PBS, and imaging was performed after adding PBS with sulforhodamine B. The lower panel contains coronal (XZ) and transverse (YZ) sections through the cells treated with BacSp222-CF488, showing the spatial distribution of the peptide

Our previous studies revealed that BacSp222-stimulated NF-κB activation, leading to increased production of NO and proinflammatory cytokines in human and murine leukocyte cell lines. Therefore, we analyzed bacteriocin interaction with P388.D1 cells to elucidate the mechanism of this phenomenon. In the first experiments, we confirmed time-dependent bacteriocin internalization to cells using fluorescently labeled BacSp222-CF488 and live-cell confocal imaging. The imaging was performed in the presence of sulforhodamine B, which did not stain the cells, indicating the intact cell membrane and, simultaneously, specific bacteriocin internalization (Fig. 1). Additionally, we confirmed that BacSp222-CF488 did not colocalize with DAPI (Supplementary Fig. 1). This observation excluded nuclear localization of BacSp222-CF488 in fixed P388.D1 cells.

### FPR1 and FPR2 antagonists do not inhibit TNF production by bacteriocin-stimulated murine macrophage-like cell line

**Fig. 2:**
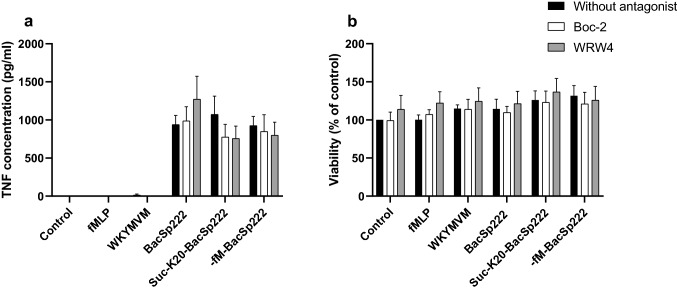
Antagonists of FPR1 and FPR2 do not block BacSp222-induced TNF expression in RAW 264.7 cells. The cells were pretreated for 30 min with WRW4 or Boc-2. Next, the cells were incubated with medium alone or were stimulated with fMLP, WKYMVM, BacSp222, suc-K20-BacSp222 or -fM-BacSp222 for 6 h. **a** TNF was determined in culture media using an ELISA test. **b** Viability of the cells was determined using a MTT method. The bars represent the mean ± SD (*n* = 3)

Our previous studies demonstrated that natural forms of BacSp222 more efficiently stimulated NF-κB activation and TNF expression than its -fM-BacSp222 form, with chemically removed formylated methionine at the N-terminus [15]. On the other hand, our separate bioluminescent assays on cAMPZen FroZen human recombinant CHO-K1 cells overexpressing FPR2 suggested that BacSp222 was not able to stimulate such receptors [20]. Therefore, to clarify this contradiction, we analyzed the effect of specific FPR antagonists (Boc-2 or WRW4, selective toward FPR1 and FPR2, respectively) on bacteriocin-induced TNF production. Before experiments, the potential antagonists’ cytotoxicity was excluded by viability analysis (Fig. 2b and Supplementary Fig. 2b). Then, RAW 264.7 cells were pretreated with Boc-2 or WRW4 for 30 min, and, after this, BacSp222, suc-K20-BacSp222, or -fM-BacSp222 were added to the media for another 6 h. Subsequent analysis of TNF concentration in the post-culture media revealed that the inhibitors did not affect bacteriocin-induced TNF expression (Fig. 2a). Similar effect was obtained for P388.D1 cells (Supplementary Fig. 2a). At the same time, FPRs’ agonists, fMLP and WKYMVM, did not stimulate TNF expression. These results clearly showed that some receptors other than FPR1 or FPR2 must be responsible for bacteriocin recognition.

### BacSp222 and its modified forms activate the human TLR2 receptor

**Fig. 3:**
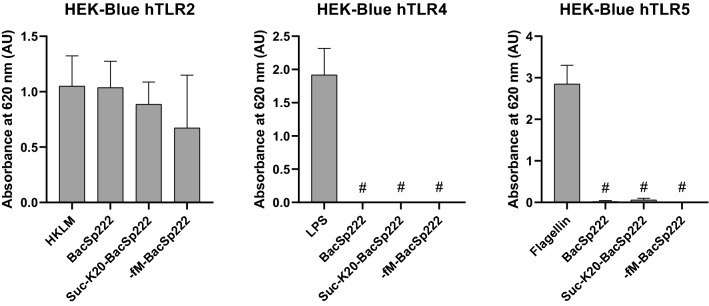
All tested forms of bacteriocin, BacSp222, suc-K20-BacSp222 and -fM-BacSp222, activate TLR2 receptor. HEK-Blue hTLR2, hTLR4 and hTLR5 cells were incubated in medium alone (negative control) or stimulated with BacSp222, suc-K20-BacSp222, -fM-BacSp222, or with positive control agonist (HKLM in case of HEK-Blue hTLR2, LPS for HEK-Blue hTLR4, or flagellin for HEK-Blue hTLR5). After this the SEAP activity was measured in culture media after colorimetric reaction with the Cell Culture Medium for SEAP reagent. The absorbance measured for negative controls (cells incubated with medium without additives) was subtracted from the absorbance measured for subsequent samples. The bars represent the mean ± SD (*n* = 3), ^#^*p* < 0.001 vs positive control

Other essential receptors responsible for recognizing PAMPs are receptors belonging to the TLR family. TLRs are a diverse group comprising 10 members in humans and 13 members in mice [21]. Based on the cellular location of such receptors, they were divided into two groups: (1) cell surface TLRs (TLR1, TLR2, TLR4–TLR6, and only in humans TLR10) and (2) intracellular TLRs (TLR3, TLR7–TLR9, and only in mouse TLR11–TLR13). Bacterial proteins and peptides are indicated as ligands only for membrane TLRs, and therefore, for our studies, we selected TLR2, TLR4, and TLR5. We used genetically modified HEK-Blue, HEK293 cells that overexpressed the selected receptor. In these cells, the binding of a specific ligand to the receptor results in NF-κB activation and expression of the reporter enzyme—secreted embryonic alkalic phosphatase—SEAP. HEK-Blue cells were stimulated overnight by the appropriate specific ligands (HKLM, LPS, or flagellin, for TLR2, TLR4, and TLR5, respectively) and by BacSp222, suc-K20-BacS222 and -fM-BacSp222. After this, the SEAP activity was colorimetrically analyzed in the post-culture media and indicated activation of the particular TLR by a specific ligand. As shown in Fig. 3, the high absorbance level was noticed only for HEK-Blue hTLR2 cells stimulated with all the tested bacteriocins. Activation of HEK-Blue hTLR4 and HEK-Blue hTLR5 was observed only in response to LPS or flagellin, respectively (Fig. 3). These observations clearly indicate that BacSp222 bacteriocin and its modified forms activate the TLR2 receptor.

### TLR2 antagonists inhibit TNF release by bacteriocin-stimulated macrophage-like cells

**Fig. 4:**
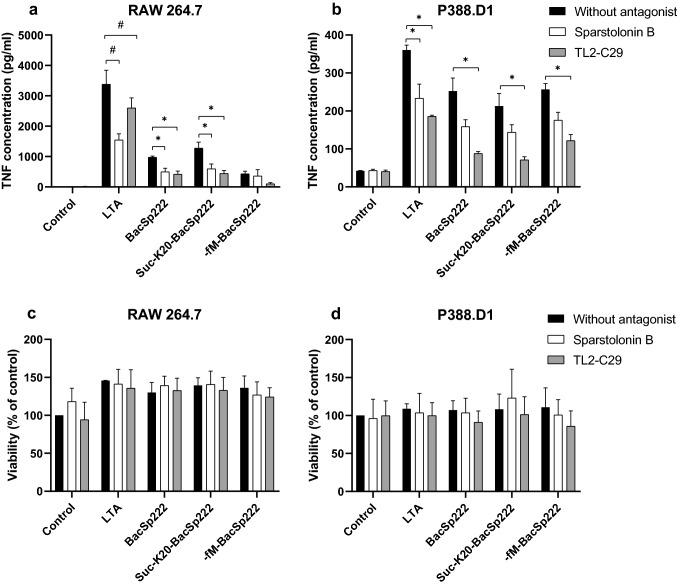
The effect of TLR2 inhibitors on BacSp222-induced TNF production by RAW 264.7 and P388.D1 cells. **a**, **b** The cells were stimulated with BacSp222, suc-K20-BacSp222, -fM-BacSp222 or LTA in media alone or in media containing sparstolonin B or TL2-C29, and the TNF concentration released to the culture media was analyzed by an ELISA test. **c**, **d** The possible toxicity of inhibitors used was excluded using the MTT test. The bars represent the mean ± SD (*n* = 3), ^#^*p* < 0.001 and *p < 0.05 was evaluated vs cells stimulated without antagonists

As we have shown above, all tested forms of BacSp222 bacteriocin were able to activate the human TLR2 receptor. Since our previously published results demonstrated that BacSp222-induced TNF expression in murine macrophage-like cell line [15], we have decided to verify if TLR2 antagonists, such as sparstolonin B or TL2-C29 [22, 23], affect the TNF production by bacteriocin-stimulated RAW 264.7 and P388.D1 cells. The cells were pretreated with mentioned antagonists for 30 min and then stimulated for 6 h with specific BacSp222 bacteriocin or LTA (the ligand for TLR2 and a positive control of the test). Subsequent analysis of TNF concentration in the culture media revealed that both sparstolonin B and TL2-C29 significantly decreased BacSp222 or suc-K20-BacSp222-induced TNF production by RAW 264.7 cells (Fig. 4a). Sparstolonin B decreased TNF secretion from 976 to 499 pg/mL (for BacSp222) and from 1279 to 599 pg/mL (for suc-K20-BacSp222). Similarly, TL2-C29 reduced TNF production to 418 pg/mL and 445 pg/mL for BacSp222 or suc-K20-BacSp222, respectively. At the same time, for P388.D1 cells, only TL2-C29 reduced the level of TNF expression after stimulation with bacteriocins in a statistically significant way (*p* < 0.05). It decreased TNF concentration from 252 to 88 pg/mL, from 213 to 71 pg/mL, and from 257 to 122 pg/mL for BacSp222, suc-K20-BacSp222 and -fM-BacSp222, respectively (Fig. 4b). The decrease in TNF secretion was also confirmed for the cells stimulated with LTA in the presence of antagonists. In all cases, the potential cytotoxicity of tested antagonists was excluded by the viability test (Fig. 4c, d).

### BacSp222 and its modified forms activate human TLR2/TLR6 heterodimers

**Fig. 5:**
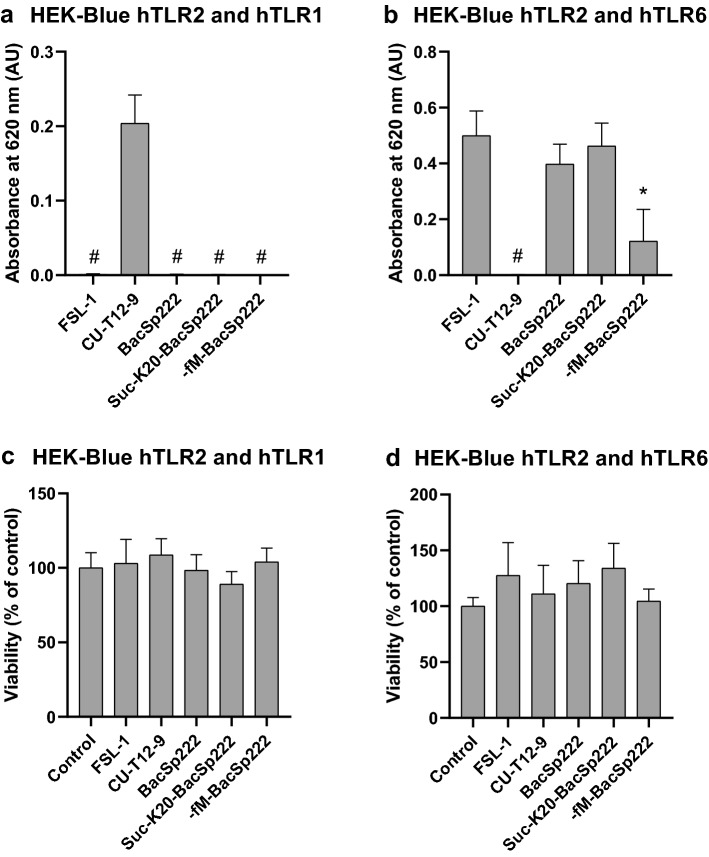
All forms of BacSp222 activate hTLR2/TLR6 heterodimers. **a** HEK-Blue hTLR2 and hTLR1or **b** HEK-Blue hTLR2 and hTLR6 were incubated with medium alone or stimulated with bacteriocins, FSL-1 (positive control for HEK-Blue hTLR2 and hTLR6) or CU-T12-9 (positive control for HEK-Blue hTLR2 and hTLR1). SEAP activity was measured colorimetrically in culture media after reaction with the cell culture medium for SEAP reagent. The absorbance measured for negative controls (the cells incubated with medium without additives) was subtracted from the absorbance measured for subsequent samples. **c**, **d** Viability of the cells was analyzed with the MTT method. The bars represent the mean ± SD (*n* = 3), ^#^*p* < 0.001 vs positive control

TLR2 recognizes especially LTAs from Gram-positive bacteria, but also particular lipopeptides, selected lipoarabinomannans and zymosans as well as atypical LPS [24]. The chemical and structural diversity of ligands recognized by TLR2 is related to the ability of this receptor to form a variety of heterodimers. According to current knowledge, TLR2 forms heterodimers mainly with TLR1 or TLR6. So, to identify the TLR2 partner for BacSp222 bacteriocin recognition, we used HEK-Blue cells overexpressing human TLR2, co-receptor CD14 and TLR1 (the cells forming TLR2/TLR1 heterodimer), or HEK-Blue cells overexpressing human TLR2, CD14, and TLR6 (the cells forming TLR2/TLR6 heterodimer). These cells were exposed overnight to BacSp222, suc-K20-BacSp222, -fM-BacSp222, or to particular TLR2 ligands: CU-T12-9 (specific for TLR2/TLR1) and FSL-1 (specific for TLR2/TLR6). Then the activity of SEAP released into the medium was measured, indicating the activation of TLR2/TLR6 or TLR2/TLR1. As shown in Fig. 5a, b, all tested forms of BacSp222 interacted only with TLR2/TLR6 heterodimers. Moreover, TLR2/TLR6 activation was lower for the -fM-BacSp222 form of bacteriocin, suggesting that the N-terminal part of the BacSp222 molecule is important for interaction with the receptor. The cells overexpressing TLR2/TLR1 heterodimers were activated by none of the tested forms of bacteriocin; TLR2/TLR1 activation was observed only after exposition to FSL-1. As shown in Fig. 5c, d, none of the tested ligands exerted cytotoxicity against studied cells.

### TL2-C29, a specific antagonist of TLR2, inhibits BacSp222-induced TLR2/TLR6 heterodimer activation

**Fig. 6:**
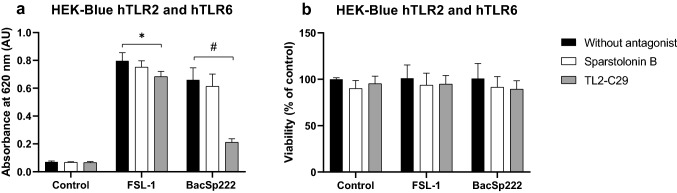
The effect of TLR2 inhibitors on BacSp222-induced activation of hTLR2/TLR6 heterodimer. **a** HEK-Blue hTLR2 and hTLR6 were activated with BacSp222 or FSL-1 (positive control) in media without antagonist or in media containing sparstolonin B or TL2-C29. SEAP activity was measured in culture media and indicates receptor stimulation. **b** The possible toxicity of inhibitors was excluded using the MTT method. The bars represent the mean ± SD (*n* = 3), ^#^*p* < 0.001 and *p < 0.05 vs cells stimulated without antagonist

Knowing that BacSp222 bacteriocin is recognized by human TLR2/TLR6 heterodimer, we verified whether TLR2 antagonists, such as sparstolonin B or TL2-C29, limit the activation of the heterodimer by bacteriocins. So, HEK-Blue cells expressing hTLR2/TLR6 heterodimers were prestimulated for 1 h with sparstolonin B or TL2-C29. Next, the cells were stimulated overnight with BacSp222 or FSL-1, and, after this, the activity of SEAP released into the medium was measured. As shown in Fig. 6, only TL2-C29 significantly (*p* < 0.001) reduced the production of SEAP, indicating inhibition of TLR2/TLR6 activation by specific ligands. This result confirms that BacSp222 specifically stimulates the TLR2/TLR6 heterodimers and that this interaction can be inhibited by TLR2 antagonists such as TL2-C29.

### Preparations of BacSp222 or suc-K20-BacSp222 are not contaminated with LTA

LTA, an essential component of the cell wall of Gram-positive bacteria, is an important virulence factor, interacting with eukaryotic cells via the TLR2/TLR6 heterodimer [25, 26]. As described earlier, BacSp222 and its post-translationally modified form, suc-K20-BacSp222, are peptides isolated from the post-culture medium of *Staphylococcus pseudintermedius* 222. In this regard, in this study, it was essential to verify thoroughly whether the bacteriocin preparations used were not contaminated with LTA. Because LTA is a heterogenic molecule, difficult to direct quantification, we decided on indirect determinations of total phosphorus in bacteriocin preparations, taking advantage of the fact that all types of LTA contain organic phosphodiester bonds, absent, in turn, in bacteriocin molecules. Performed measurements showed that the maximal concentration of LTA detectable in 1 µM working bacteriocin preparation is 0.011 µg/mL (Supplementary table 1), and this amount was three times lower than the lowest concentration of LTA, which was able to stimulate RAW 264.7 cells to produce NO in co-stimulation with IFN (Supplementary Fig. 3). These results clearly prove that used in our study BacSp222 and suc-K20-BacSp222 preparations are not contaminated with significant amounts of LTA and that the observed by us TLR2/TLR6 activation phenomenon is caused solely by tested bacteriocins. Moreover, the exclusion of bacteriocin contamination by LTA using a phosphorus method additionally confirmed also lack of LPS in bacteriocin preparations, independently verified in our previous work by a specific endotoxin detection kit [15]. This is because both LTA as well as LPS contain phosphate groups.

## Discussion

The number of bacteria cells living in the average human body is roughly equal to the number of somatic cells and almost each bacterial species can produce at least one kind of bacteriocin [27]. These molecules appear to be critical factors influencing the proper microbiome composition of the skin, mucous membranes, as well as gastrointestinal and urogenital tracts [11]. The current interest in bacteriocins is mainly related to their potential use as antibacterial compounds and antibiotic substitutes. However, the impact of bacteriocins on human or animal physiology, especially on the host's immune system, is poorly documented.

Our previous studies showed that BacSp222 bacteriocin and its succinylated forms exhibit significant proinflammatory properties [15]. Therefore, in the present study, we aimed to elucidate the mechanism of bacteriocin recognition by immune system cells. First, we observed that BacSp222 undergoes internalization into P388.D1 cells without damaging membrane integrity. So far, the direct interaction of other bacteriocins with eucaryotic cells, remarkably immune cells, has been poorly documented. For example, Martínez-García and colleagues showed that FITC-labeled bacteriocin AS-48 is endocytosed into *Trypanosoma brucei* depending on clathrin and temperature. However, the observed endocytosis was closely related to the formation of autophagic vacuoles and, consequently, to the toxic effect of the tested bacteriocin on the protozoan [28]. In contrast, Dreyer et al. showed that nisin, plantaricin 423, and bacST4SA labeled with NHS fluorescein, used in non-toxic concentrations, could bind to the cell membrane or penetrate Caco-2 epithelial cells and human umbilical vascular endothelial cells (HUVEC). However, these studies did not specify the exact cellular location of these bacteriocins [29].

Next, we aimed to identify receptors involved in BacSp222 recognition by host cells and mediating intracellular signals leading to stimulation of inflammatory response. Bacteriocin BacSp222 is a peptide secreted into the environment by the opportunistic strain of *Staphylococcus pseudintermedius* 222 [14]. Therefore, the most likely receptor responsible for recognizing BacSp222 seemed to be a receptor belonging to the pattern recognition receptors (PRRs). Since our previous observations indicated that bacteriocin BacSp222 with chemically cleaved formylated methionine exerted lower proinflammatory activity than its natural forms, BacSp222, or suc-K20-BacSp222 [15], we first focused on receptors belonging to FPRs (specifically FPR1 or FPR2) and known to mediate intracellular signaling after binding peptides containing formylated methionine at the N-terminus [30]. However, as was stated in the Results section, our separate bioluminescent assays on cAMPZen FroZen human recombinant CHO-K1 cells overexpressing FPR2 suggested that BacSp222 was not able to stimulate such receptors [20]. To confirm such results, in the present study, we observed that specific FPR antagonists Boc-2 (antagonist for FPR1, at higher concentrations also antagonist for FPR2 [31]) and WRW4 (antagonist for FPR2 [32]) did not affect bacteriocin-induced TNF production by RAW 264.7 and P388.D1 cells. Therefore, based on these observations, we excluded FPRs as receptors involved in BacSp222 bacteriocin recognition.

Another family of PRR receptors activated during the first stages of microbial infection is the TLR family. These receptors are expressed on all innate immune cells [33]. Their ligands are various molecules that differ in hydrophobicity, size, and structure, including different proteins, lipids, and nucleic acids [34]. TLRs molecules comprise extracellular leucine-rich repeats (LRR) domain, responsible for ligand recognition, transmembrane domain, and intracellular toll–interleukin 1 receptor (TIR) domain, involved in signal transmission in the cell [35]. TLRs expressed on the cell surface primarily recognize proteins, peptidoglycans, and lipopeptides, while intracellular TLRs recognize predominantly nucleic acids [36]. In our research, we used genetically modified HEK-Blue cells that overexpressed the most crucial human cell surface TLRs: TLR2, TLR4, and TLR5. These cells were not devoid of any endogenous receptor. We observed that TLR2 was the only receptor activated after exposure to all tested forms of BacSp222, including its deformylated form, -fM-BacSp222. Although TLR2 is a membrane receptor, its endocytosis is necessary for the cell's response to LTA [37]. Moreover, activation of the NF-κB-dependent signaling pathway by human THP-1 monocytes or human TLR2-overexpressing HEK-Blue reporter cells stimulated with LTA or Pam3CSK4 requires receptor-dependent endocytosis, and inhibition of this process affects the level of TNF released by the cells [38]. We believe that the internalization of BacSp222 by P388.D1 cells, shown in this study, is most likely just a consequence of endocytosis of the TLR2-BacSp222 complex.

The central role of TLR2 is to recognize infection with Gram-positive bacteria. The ligands for this receptor are mainly LTAs, a major constituents of the Gram-positive bacteria cell wall. TLR2 also recognizes lipopeptides, atypical LPS, as well as selected lipoarabinomannans and zymosans [24]. Such a variety of recognized ligands is related to the fact that TLR2 can also form heterodimers, e.g., with TLR1, TLR6, and, in humans, TLR10 [39]. Our observations indicated that BacSp222 is recognized by both human and murine cells. Therefore, in the further stage of our research, we focused exclusively on the interaction between bacteriocin and TLR2/TLR1 as well as TLR2/TLR6 heterodimers. We used HEK-Blue cells with deleted endogenous TLR1 and TLR6, which expressing exogenous human heterodimers TLR2/TLR1 or TLR2/TLR6. Bacteriocin and its modified forms activated only TLR2/TLR6 heterodimer, indicating that TLR6 is indispensable for signal transmission. Therefore, our results clearly demonstrated that the studied bacteriocin is a novel ligand for the TLR2/TLR6 heterodimer. According to the published data, known ligands for this heterodimer are LTA [25, 26], diacylated lipopeptides (e.g. mycoplasmal macrophage activating lipopeptide-2 [40]) and zymosan [41]. At the same time, for the TLR2/TLR1 heterodimer, several protein ligands have been described, such as the B pentamer heat-labile enterotoxin, produced by *Escherichia coli* [42], or the BspA protein, produced by *Tannerella forsythia* [43].

Of course, the chemical nature of BacSp222 is fundamentally different from mentioned known typical TLR2/TLR6 ligands—namely LTA. Therefore, it was crucial in our work to prove that such a compound did not contaminate the studied bacteriocin preparations. LTA is a complex polymer formed of repeating units of polyhydroxy alkanes, glycerol, and ribitol, joined via phosphodiester linkages and anchored by a lipid moiety to the membrane of Gram-positive bacteria. LTA molecules have been grouped into different types, assuming the chemical nature of substituents decorating polyglycerol-phosphate subunits, the length of the whole polymer, and the nature of the glycolipid anchor in the cellular membrane [44, 45]. Such a chemical diversity makes the quantitative analyses of LTAs very difficult. Therefore, we decided on indirect determinations of total phosphorus in bacteriocin preparations, taking advantage of the fact that all types of LTA contain organic phosphodiester bonds, absent, in turn, in molecules of the peptides studied. The results of such analyses confirmed that all peptides used in our study lack phosphorus and, thus, also possible LTA contamination. Therefore, observed TLR2/TLR6 activation is a straightforward consequence of bacteriocin recognition and binding. However, further studies are necessary to elucidate what specific part of the bacteriocin molecule is responsible for interacting with the TLR2/TLR6 heterodimer.

In subsequent experiments, we verified whether TLR2 antagonists (sparstolonin B, and TL2-C29) could inhibit the interaction of bacteriocin with the receptor. Both these antagonists have a similar mechanism of action, which is associated with limiting the interaction of MyD88 with the TIR subunit of TLR2 (and for sparstolonin B also TLR4) [22, 23]. As described in the Results section, sparstolonin B did not reduce the level of TNF released in all stimulation variants performed on RAW 264.7 or P388.D1 cells. Whereas, TL2-C29 affected TNF levels in virtually every tested sample. However, the lack of TLR2/TLR6 inhibition by sparstolonin B is most likely related to its low concentration in our experiments (6 µM for RAW 264.7 and P388.D1 cells, 20 µM for HEK-Blue cells). Liang et al*.* tested on RAW 264.7 cells the inhibitory effect of sparstolonin B in 10 µM or 100 µM concentrations [46], but in our study, these concentrations were toxic to RAW 264.7 cells (data not shown). Mistry et al*.* showed that TL2-C29 inhibits the signaling of human TLR2/TLR6 and TLR2/TLR1 heterodimers and murine TLR2/TLR1 heterodimer [23]. However, our studies on RAW 264.7 and P388.D1 cells showed that TL2-C29 inhibited the interaction of bacteriocin with human TLR2/TLR6 and the murine TLR2/TLR6. Our results align with the results shared by the TL2-C29 manufacturer (InvivoGen web page, https://www.invivogen.com/tlr2-inh-c29, accessed on 02.02.2023) and indicate that TL2-C29 is also an antagonist for the murine TLR2/TLR6 and, in higher concentrations, TLR2/TLR1 dimer. Moreover and importantly, inhibition of BacSp222-stimulated TLR2/TLR6 signaling by sparstolonin B and TL2-C29 indicates that the adapter protein MyD88 is involved in the signaling cascade. Summarizing, our studies revealed the TLR2/TLR6/MyD88/NF-κB pathway as a potential mechanism explaining the inflammatory response activated by BacSp222 bacteriocin in human and murine cells.

The primary limitation of the other reports documenting the influence of different bacteriocins on the immune response of the host or inflammatory reactions is the usage of insufficiently pure peptide preparations, especially those not verified for endotoxins content. To date, only five peptide bacteriocins have been reliably verified for their activity toward eukaryotic cells: microcin J25 [47–49], nisin [50, 51], pyocin S5 [52], as well as avicin A and acidocin A [53]. All these reports concerned carefully purified peptides, and the studies were performed in vitro, using different cell lines, and/or in vivo, on mouse, rat, or *Galleria mellonella* animal models. The researchers verified mainly the influence of studied peptides on inflammatory reactions. And so, three bacteriocins, microcin J25, nisin, and pyocin S5, revealed evident anti-inflammatory properties. On the other hand, two pediocin-like bacteriocins, avicin A and acidocin A, demonstrated different proinflammatory activity, manifested by an increase in the production of proinflammatory cytokines and chemokines by human primary monocytes [53]. However, the mechanism of such a phenomenon is, to date, puzzling. Kindrachuk et al*.* studied signaling pathways activated by nisin and showed that this bacteriocin stimulated the phosphorylation of p38 mitogen-activated protein kinases (p38 MAPKs), Akt serine/threonine kinase (Akt), and cAMP-responsive element binding protein (CREB) in human primary monocytes [51]. Although such work is intriguing, it contains some shortcomings, making it difficult to draw clear conclusions concerning the mechanism of nisin activity toward studied cells. We believe the present study is the first to identify a specific eukaryotic cell receptor responsible for recognizing the proinflammatory bacteriocin molecule.

## Supplementary Information

Below is the link to the electronic supplementary material.

Supplementary file1 (PDF 552 KB)

## Data Availability

The authors declare that all relevant data generated or analyzed during this study are included in this published article and its supplementary information files.
