# Supplementary material for: BacSp222 bacteriocin as a novel ligand for TLR2/TLR6 heterodimer
Source: Inflamm Res. 2023 Mar 25;72(5):915–28. doi: 10.1007/s00011-023-01721-3 (PMC10198859; doi:10.1007/s00011-023-01721-3)
Supplement: Supplementary file 1 — Supplementary file1 (PDF 552 KB) [file 11_2023_1721_MOESM1_ESM.pdf]

Supplementary information

Justyna Śmiałek-Bartyzel[1, 2], Monika Bzowska[3], Renata Mężyk-Kopeć[3], Marcin Kwissa[4], Paweł Mak[2, ✉]

BacSp222 bacteriocin as a novel ligand for TLR2/TLR6 heterodimer

[1] Jagiellonian University, Doctoral School of Exact and Natural Sciences, Łojasiewicza 11 St., 30-348 Kraków, Poland.

[2] Jagiellonian University, Faculty of Biochemistry, Biophysics and Biotechnology, Department of Analytical Biochemistry, Gronostajowa 7 St, 30-387 Kraków, Poland.

[3] Jagiellonian University, Faculty of Biochemistry, Biophysics and Biotechnology, Department of Cell Biochemistry, Gronostajowa 7 St, 30-387 Kraków, Poland.

[4] Pritzker School of Molecular Engineering, University of Chicago, 5640 South Ellis Ave., Chicago, IL 60637, United States.

✉ pawel.mak@uj.edu.pl, phone +48 12 6646511, fax +48 12 6646915

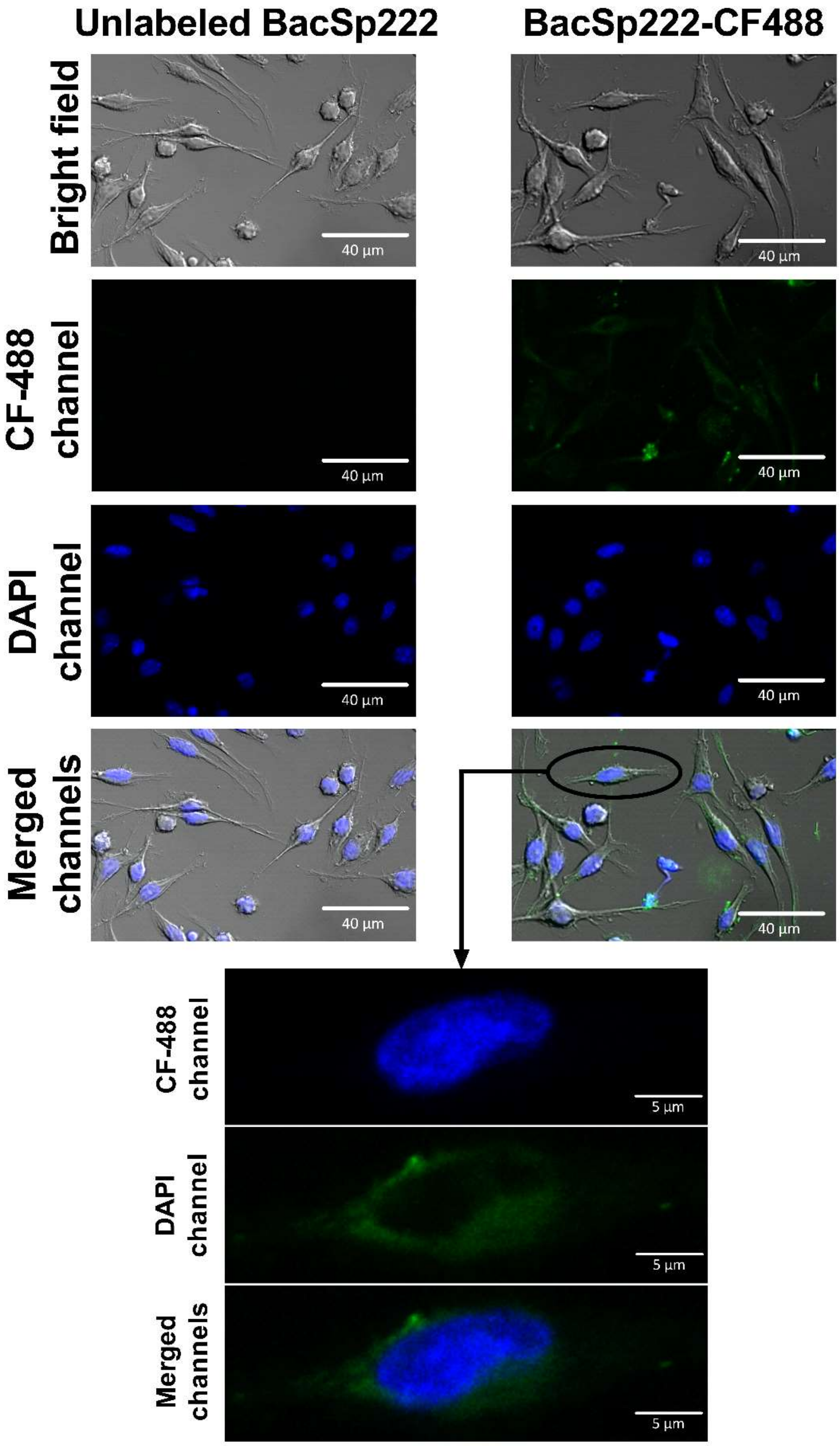

Supplementary figure 1. Bac-Sp222-CF488 does not colocalize with the nucleus of P388.D1 cells. BacSp222 or BacSp222-CF488 were added to the cells for 30 minutes. After incubation, the cells were washed with PBS, and fixed by methanol-free formaldehyde, then the nuclei were stained with DAPI. All visualizations were made using confocal microscopy.

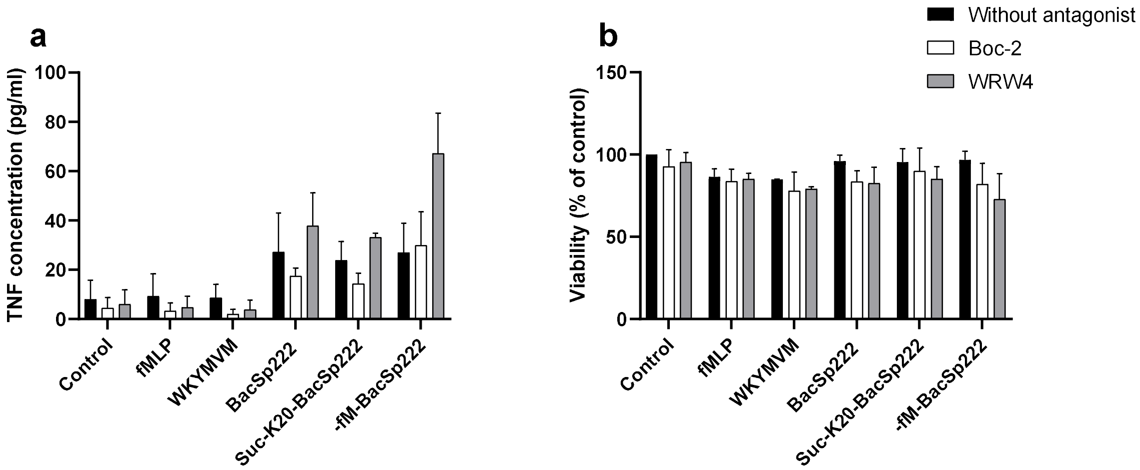

Supplementary figure 2. Antagonists of FPR1 and FPR2 do not block BacSp222-induced TNF expression in P388.D1 cells. The cells were pretreated for 30 minutes with WRW4 or Boc-2 and then stimulated with fMLP, WKYMVM, BacSp222, suc-K20-BacSp222 or -fM-BacSp222 for 6 hours. **a** TNF was determined in culture media using an ELISA test. **b** Viability of the cells was determined using a MTT method. The bars represent the mean ± SD (n=3).

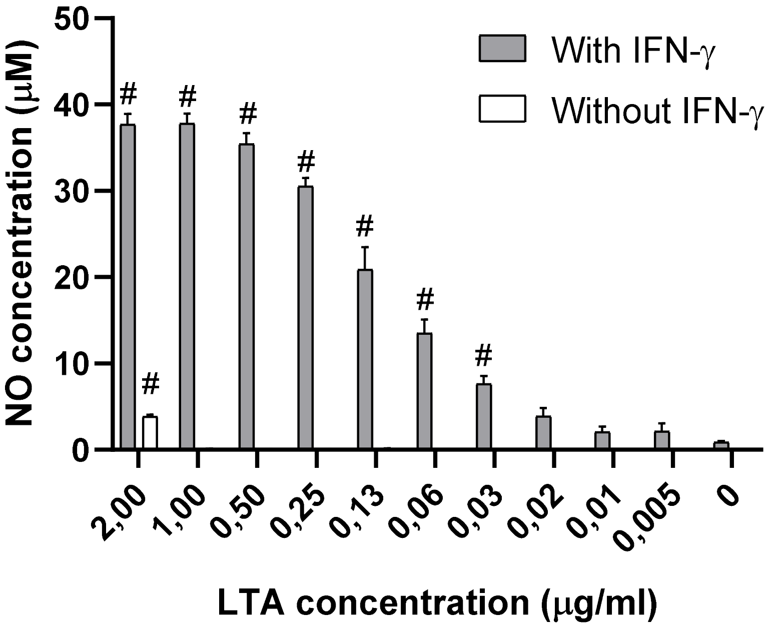

Supplementary figure 3. LTA enhances IFN-γ induced NO production by iNOS in RAW 264.7 cells. The cells were cultured with alone medium or were stimulated for 24 h in different LTA concentrations in absence or presence of IFN-γ. Then the nitrate level secreted to the culture media was measured using the Griess reaction. The bars represent the mean ± SD (n=3), #p<0.001 vs cells not stimulated by LTA.

Supplementary table 1. Both BacSp222 and suc-K20-BacSp222 preparations do not contain significant amounts of LTA.

Sample	Batch	Repetition	Amount of LTA (µg) detected in 1 ml 1 µM solution of the peptide
BacSp222	1	1	<0,0041
		2	<0,0041
		3	0,0101
	2	1	0,0110
		2	<0,0041
		3	0,0085
	3	1	<0,0041
		2	<0,0041
		3	0,0076
Suc-K20-BacSp222	1	1	<0,0041
		2	<0,0041
		3	<0,0041
	2	1	0,0110
		2	0,0093
		3	0,0093
	3	1	<0,0041
		2	<0,0041
		3	<0,0041
